# The relationship between anaesthetic technique, clinicopathological characteristics and the magnitude of the postoperative systemic inflammatory response in patients undergoing elective surgery for colon cancer

**DOI:** 10.1371/journal.pone.0228580

**Published:** 2020-04-29

**Authors:** Aliah M. Alhayyan, Stephen T. McSorley, Rachel J. Kearns, Paul G. Horgan, Campbell S. D. Roxburgh, Donald C. McMillan

**Affiliations:** 1 College of Medical, Veterinary and Life of Sciences, School of Medicine–University of Glasgow, Glasgow, United Kingdom; 2 School of Medicine, Dentistry & Nursing–University of Glasgow, Glasgow, United Kingdom; 3 Department of Anaesthetics, School of Medicine, Dentistry & Nursing–University of Glasgow, Glasgow, United Kingdom; 4 Institute of Cancer Sciences, School of Medicine, Dentistry & Nursing–University of Glasgow, Glasgow, United Kingdom; 5 School of Medicine, Dentistry & Nursing–University of Glasgow, Glasgow, United Kingdom; 6 Institute of Cancer Sciences, Department of Surgery, School of Medicine, Dentistry & Nursing–University of Glasgow, Glasgow, United Kingdom; Melbourne University, AUSTRALIA

## Abstract

**Background/aim:**

The magnitude of the postoperative systemic inflammatory response (SIR) is now recognised to be associated with both short and long-term outcomes in patients undergoing surgery for colon cancer. During such surgery, it is unclear whether the anaesthetic regimens influence the magnitude of the postoperative SIR, independent of other factors. The aim of the present study was to examine the association between anaesthetic agents, clinicopathological characteristics and the magnitude of the postoperative SIR in patients undergoing elective surgery for colon cancer.

**Methods:**

Patients with colon cancer who underwent elective open or laparoscopic surgery between 2008 and 2016 (n = 409) were studied at a single center. The relationship between type of anaesthesia, surgical technique; open (n = 241) versus laparoscopic (n = 168) and clinicopathological characteristics was examined by using chi-square testing. The chi-square test was used to determine which anaesthetic group influences the POD 2 CRP for only patients undergoing elective open colon surgery.

**Results:**

The majority of patients were <75 years old, male, normal weight or obese, underwent open surgery and had regional anaesthesia, in particular an epidural approach. There was a significant association between type of anaesthesia and post-operative CRP on day 2 (p <0.001) in patients undergoing open surgery but not laparoscopic surgery. Other factors associated with type of anaesthesia included; year of operation (p <0.01), surgical technique (p <0.001), and preoperative dexamethasone (p <0.01).

**Conclusion:**

In patients undergoing surgery for elective colon cancer, the type of anaesthesia varied over time. The type of anaesthesia appears to influence the magnitude of the postoperative SIR on post-operative day 2 in open surgery but not laparoscopic surgery. Future work using prospective study design is required to better define this relationship.

## 1. Introduction

Surgical resection remains the mainstay of treatment for patients with non-metastatic solid tumours. However, the magnitude of the stress response from surgical injury may lead to alterations in the immune function, neuroendocrine and metabolic responses and in turn may instigate the progression and recurrence of cancer [1].

Routinely, the magnitude of the post-operative SIR, is evidenced by C-reactive protein (CRP) concentration in the blood [2, 3]. In turn, the magnitude of the post-operative CRP response has been shown to be associated with post-operative complications [4]. More recently, a threshold of a CRP >150 mg/L on day 3 or day 4 has been shown to be associated with the development of post-operative complications and greater hospital stay [5]. With the establishment of a post-operative CRP threshold, potential factors giving rise to an elevated post-operative CRP are being increasingly identified in operable colorectal cancer. To date the pre-operative factors identified to independently modulate the SIR following surgery include age, ASA grade, BMI, pre-operative modified Glasgow Prognostic Score (mGPS) and most recently preoperative corticosteroids [4, 6] and these should be incorporated into any analysis of the effect of anaesthesia.

Recently, a systematic review and meta-analysis by Alhayyan and co-workers, reported that due to the heterogeneity of previous studies, it is not clear whether different anaesthetic approaches modulate the magnitude of the post-operative SIR as evidenced by IL-6 and CRP [7, 8]. However, the systematic review was not able to account for a number of potential confounding factors, in particular the type of surgery since open and laparoscopic surgical techniques are recognised to be associated with a different magnitude of the SIR [3, 9].

Regional anaesthesia is an integral component of enhanced recovery programmes which aim to; reduce the perioperative neural and hormonal stress responses, manage pain, optimise post-operative mobilisation, aid return to oral nutrition, and facilitate recovery. The provision of multi-modal, balanced analgesia has the advantage of reducing opioid consumption and associated adverse effects. Whilst epidural analgesia was traditionally considered the gold standard for analgesia in patients undergoing open colorectal surgery, the evolution of minimally invasive surgery in combination with alternative analgesic techniques such as intrathecal opioid administration, abdominal wall blocks, continuous wound infusions and intravenous lignocaine now forms a central component of most accelerated surgical pathways [10].

The aim of the present study was to examine the association between different anaesthetic technique, clinicopathological characteristics and the magnitude of the post-operative SIR in patients undergoing elective surgery for colon cancer.

## 2. Patients and methods

### 2.1 Patients

A prospective database consisted of 543 patients who underwent for elective open or laparoscopic colon cancer resection was retrospectively reviewed in a single surgical unit at Glasgow Royal Infirmary hospital between 2008 and 2016. The total number of patients who had documented anaesthetic regimen was 409; for either open (n = 241) or laparoscopic approach (n = 168). Only 61 patients received general anaesthesia alone with the remaining 348 receiving general anaesthesia plus a regional anaesthetic technique. Regional anaesthesia was subdivided into four subgroups; (general plus epidural (GA+E) n = 156; general plus spinal opioid (GA+Sp) n = 91; general plus Transversus Abdominus Plane block (GA+TAP) n = 60; general plus local anaesthetic infiltration (GA+LA) n = 41.

### 2.2 Methods

All data were anonymised, and the emergency cases were excluded from the analysis. All tumours were staged according to TNM staging system (tumour, node and metastasis). The American Society of Anaesthesiologists (ASA) grading system was used to assess patient comorbidity [11]. The modified Glasgow Prognostic Score (mGPS) from 0–2, was used to assess the preoperative systemic inflammatory response. Patients with normal CRP concentration (<10 mg/L) scored zero. Patients with high CRP concentration (>10 mg/L) scored 1 and patients with high CRP concentration (>10 mg/L) and hypoalbuminaemia (<35 g/L) scored 2 [12]. The measurement of post-operative C-reactive protein (CRP) on the second, third and fourth day was used to assess the magnitude of the postoperative SIR.

Patients data were collected from a prospective database from January to December 2016 from the academic department of surgery at Glasgow Royal Infirmary hospital. The study was approved by the West of Scotland Research Ethics Committee, Glasgow.

## 3. Data analysis

All data were analysed using SPSS version 25.0 for windows (IBM Corporation, Armonk, NY, USA). Analysis of frequency was used to calculate the total numbers of each explanatory variable. The X^2^ (Chi-square) statistical method was used to test the statistical significance between the anaesthetic agents, surgical technique and clinicopathological variables. The chi square test was used to examine which anaesthetic group differ significantly on the POD 2 CRP in patients undergoing elective open colon surgery. A p-value of <0.05 was considered statistically significant.

## 4. Results

The clinicopathological data of 409 patients who underwent for elective open or laparoscopic colon cancer surgery were summarized in Table 1. The year of operation was divided into two periods; 2008–2012, (n = 149) and 2013–2016, (n = 260). Patients were divided into two main groups; open surgery (n = 241) and laparoscopic surgery (n = 168). Patients received either general anaesthesia alone or general anaesthesia plus a regional anaesthetic technique. Preoperative dexamethasone was administered to 104 patients (43%) undergoing open surgery and 129 patients (76%) undergoing laparoscopic surgery.

**Table 1:** Demographic characteristics for patients undergoing surgery for elective colon cancer and the comparison between open and laparoscopic surgery for different anaesthetic groups (n = 409).

Characteristic	Number of patients (%)	Open surgery	Laparoscopic surgery	p-value
Age (<65/65-74/>75)	125 (31)/163 (40)/121 (29)	98 (31)/122 (38)/96 (30)	62 (31)/81 (41)/55 (28)	0.68
Sex (male/female)	220 (54)/189 (46)	169 (53)/147 (46)	108 (54)/90 (45)	0.44
BMI (<20/20-25/26-30/>30)	24 (6)/122 (31)/115 (29)/133 (34)	25 (9)/101 (35)/75 (26)/83 (29)	10 (5)/50 (26)/62 (32)/72 (37)	0.005
Year of operation (2008-2012/ 2013–2016)	149 (36)/260 (64)	159 (50)/157 (50)	68 (34)/130 (66)	<0.001
ASA grade (1/2/3/4)	77 (19)/185 (46)/129 (32)/11 (3)	50 (16)/124 (41)/115 (38)/15 (5)	39 (20)/95 (50)/53 (28)/4 (2)	0.007
TNM stage (I/II/III/IV)	89 (22)/166 (41)/135 (34)/13 (3)	60 (19)/128 (42)/101 (33)/18 (6)	50 (26)/71 (37)/69 (36)/2 (1)	0.08
Preop mGPS (0/1/2)	291 (78)/39 (10)/45 (12)	207 (74)/25 (9)/47 (17)	141 (81)/22 (12)/11 (6)	0.01
Surgical technique (open/laparoscopic)	241 (59)/168 (41)	-	-	-
Anaesthetic approach (G/G +E/G +TAP-B/G + LI/G +S)	61 (15)/156 (38)/60 (15)/41 (10)/91 (22)	23 (9)/144 (59)/22 (9)/11 (4)/42 (17)	38 (23)/12 (7)/38 (23)/30 (18)/49 (29)	<0.001
POD 2 CRP > 150 mg/L (no/yes) ^a^	182 (50)/181 (50)	97 (36)/171 (64)	111 (66)/57 (34)	<0.001
POD 3 CRP > 150 mg/L (no/yes)	215 (56)/172 (44)	132 (43)/172 (56)	115 (64)/63 (35)	<0.001
POD 4 CRP > 150 mg/L (no/yes) ^b^	217 (67)/106 (33)	169 (60)/113 (40)	91 (69)/41 (31)	0.04
Dexamethasone (no/yes)	177 (43)/231 (57)	137 (57)/104 (43)	40 (24)/129 (76)	<0.001
Any complication (no/yes)	251 (62)/156 (38)	159 (54)/137 (46)	134 (71)/55 (29)	<0.001

*ASA* American Society of Anaesthesiology Grading system; *BMI* body mass index; *CRP* C-reactive protein; *TNM* Tumour Node Metastases; *Preop mGPS* preoperative modified Glasgow Prognostic score; *POD* postoperative day

^a^ n = 363

^b^ n = 323, *G* General anaesthesia; *E* Epidural anaesthesia; *TAP-b* TAP-block; *LI* Local infiltration; S Spinal anaesthesia.

The majority of patients were younger than 75 years old (40%), male (54%), were normal weight or obese (65%) and underwent open surgery (59%) for colon cancer resection. Regional anaesthesia was administered in 85% of patients, with epidural the most commonly performed technique (38%). Most patients were not systemically inflamed prior to surgery mGPS (78%) and had a CRP <150 mg/L on day 3 (56%) and 4 (67%) following surgery.

The comparison between open versus laparoscopic surgery, anaesthetic technique and clinicopathological data of patients undergoing elective colon cancer surgery is shown in Table 1. There was a significant association between surgical approach, anaesthetic technique (p <0.001), BMI (p <0.01), ASA grade (p <0.01), POD 2 CRP > 150 (p <0.001), POD 3 CRP > 150 (p <0.001), POD 4 CRP > 150 (p <0.05), preoperative dexamethasone (p <0.001) and overall complications (p <0.001).

The relationship between anaesthetic technique and clinicopathological data of patients undergoing elective open surgery for colon cancer is shown in Table 2. There was a significant association between anaesthetic agents and POD 2 CRP > 150 (p <0.001), year of operation (p <0.01), and preoperative dexamethasone (p <0.01).

**Table 2:** The relationship between anaesthetic techniques and clinicopathological variables of patients undergoing elective open surgery for colon cancer (n = 241).

Characteristic	Anaesthetic agents					
	General alone (n = 23)	GA + Epidural (n = 143)	GA + TAP–block (n = 22)	GA + Local- infiltration (n = 11)	GA + Spinal (n = 42)	P-value
Age (<65/65-74/>75)	6 (26)/9 (39)/8 (35)	49 (34)/57 (40)/37 (26)	8 (36)/6 (27)/8 (36)	2 (18)/2 (18)/7 (63)	10 (24)/18 (43)/15 (33)	0.13
Sex (male/female)	10 (43)/13 (56)	80 (56)/63 (44)	9 (41)/13 (59)	2 (18)/9 (82)	26 (62)/16 (38)	0.77
BMI (<20/20-25/26-30/>30)	0 (0)/13 (56)/2 (9)/8 (35)	10 (7)/40 (31)/41 (31)/39 (30)	4 (18)/8 (36)/4 (18)/6 (27)	1 (9)/2 (18)/3 (27)/5 (45)	2 (5)/13 (32)/15 (36)/11 (27)	0.69
Year of operation (2008-2012/2013-2016)	0 (0)/23 (100)	79 (55)/64 (45)	12 (54)/10 (45)	7 (63)/4 (36)	4 (9)/38 (90)	0.005
ASA grade (1/2/3/4)	7 (30)/10 (43)/5 (22)/1 (4)	24 (17)/62 (44)/52 (36)/4 (3)	5 (23)/8 (36)/9 (41)/0 (0)	1 (9)/5 (45)/4 (36)/1 (9)	7 (18)/18 (46)/12 (31)/2 (5)	0.53
TNM stage (I/II/III/IV)	5 (23)/9 (41)/8 (36)/0 (0)	27 (19)/63 (45)/43 (31)/7 (5)	3 (13)/14 (63)/4 (18)/1 (4)	0 (0)/4 (36)/7 (63)/0 (0)	12 (29)/15 (37)/10 (24)/4 (10)	0.84
Preop mGPS (0/1/2)	16 (73)/2 (9)/4 (18)	99 (75)/12 (9)/21 (16)	15 (79)/0 (0)/4 (21)	9 (90)/0 (0)/1 (10)	30 (75)/4 (10)/6 (15)	0.68
POD 2 CRP > 150 mg/L (no/yes)	5 (25)/15 (75)	42 (32)/88 (68)	8 (42)/11 (58)	6 (67)/3 (33)	22 (59)/15 (40)	<0.001
POD 3 CRP > 150 mg/L (no/yes)	12 (57)/9 (43)	61 (44)/78 (56)	10 (45)/12 (54)	8 (80)/2 (20)	21 (51)/20 (49)	0.32
POD 4 CRP > 150 mg/L (no/yes)	13 (72)/5 (28)	81 (64)/45 (36)	12 (60)/8 (40)	7 (87)/1 (12)	25 (66)/13 (34)	0.78
Dexamethasone (no/yes)	10 (45)/12 (54)	96 (67)/47 (33)	9 (41)/13 (59)	7 (63)/4 (36)	15 (36)/27 (64)	0.006
Any complication (no/yes)	11 (50)/11 (50)	80 (56)/63 (44)	14 (67)/8 (36)	11 (100)/0 (0)	16 (39)/25 (61)	0.48

*ASA* American Society of Anaesthesiology Grading system; *BMI* body mass index; *CRP* C-reactive protein; *TNM* Tumour Node Metastases; *Preop mGPS* preoperative modified Glasgow Prognostic score; *POD* postoperative day.

The relationship between anaesthetic technique and clinicopathological data of patients undergoing elective laparoscopic surgery for colon cancer is shown in Table 3. There were no significant associations between anaesthetic agents and clinicopathological characteristics including year of operation (p = 0.99), preoperative dexamethasone (p = 0.70) and POD 2 CRP (p = 0.62).

**Table 3:** The relationship between anaesthetic techniques and clinicopathological variables of patients undergoing elective laparoscopic surgery for colon cancer (n = 168).

Characteristic	Anaesthetic agents					
	General alone (n = 38)	GA + Epidural (n = 13)	GA + TAP-block (n = 38)	GA + Local- infiltration (n = 30)	GA + Spinal (n = 49)	P-value
Age (<65/65-74/>75)	10 (26)/17 (45)/11 (29)	4 (31)/4 (31)/5 (38)	17 (45)/12 (32)/9 (24)	6 (20)/14 (47)/10 (33)	13 (26)/24 (49)/12 (24)	0.83
Sex (male/female)	20 (53)/18 (47)	12 (92)/1 (7)	20 (53)/18 (47)	15 (50)/15 (50)	26 (53)/23 (47)	0.46
BMI (<20/20-25/26-30/>30)	1 (2)/15 (39)/10 (26)/12 (32)	1 (8)/0 (0)/1 (8)/11 (84)	0 (0)/10 (26)/13 (34)/15 (39)	1 (3)/8 (27)/10 (33)/11 (37)	4 (8)/13 (27)/16 (33)/15 (31)	0.44
Year of operation (2008-2012/2013-2016)	2 (5)/36 (95)	6 (46)/7 (54)	21 (55)/17 (45)	11 (37)/19 (63)	7 (14)/42 (86)	0.99
ASA grade (1/2/3/4)	10 (26)/16 (42)/11 (29)/1 (2)	0 (0)/7 (54)/6 (46)/0 (0)	9 (24)/19 (50)/10 (26)/0 (0)	3 (10)/15 (52)/10 (34)/1 (3)	11 (23)/25 (53)/10 (21)/1 (2)	0.81
TNM stage (I/II/III/IV)	8 (21)/15 (39)/15 (39)/0 (0)	6 (46)/4 (31)/3 (23)/0 (0)	7 (18)/17 (45)/14 (37)/0 (0)	5 (17)/10 (33)/14 (47)/1 (3)	16 (33)/15 (31)/17 (35)/0 (0)	0.92
Preop mGPS (0/1/2)	30 (81)/3 (8)/4 (11)	6 (60)/2 (20)/2 (20)	28 (87)/4 (12)/0 (0)	23 (82)/4 (14)/1 (7)	35 (78)/8 (18)/2 (4)	0.58
POD 2 CRP > 150 mg/L (no/yes)	23 (68)/11 (32)	5 (38)/8 (61)	24 (75)/8 (25)	19 (68)/9 (32)	28 (68)/13 (32)	0.62
POD 3 CRP > 150 mg/L (no/yes)	24 (69)/11 (31)	8 (61)/5 (38)	24 (67)/12 (33)	20 (77)/6 (23)	27 (61)/17 (39)	0.82
POD 4 CRP > 150 mg/L (no/yes)	14 (64)/8 (36)	9 (82)/2 (18)	15 (65)/8 (35)	17 (89)/2 (10)	24 (63)/14 (37)	0.90
Dexamethasone (no/yes)	8 (21)/30 (79)	7 (54)/6 (46)	7 (18)/31 (82)	7 (23)/23 (77)	11 (22)/38 (78)	0.70
Any complication (no/yes)	30 (79)/8 (21)	7 (54)/6 (56)	30 (79)/8 (21)	24 (80)/6 (20)	28 (57)/21 (43)	0.10

*ASA* American Society of Anaesthesiology Grading system; *BMI* body mass index; *CRP* C-reactive protein; *TNM* Tumour Node Metastases; *Preop mGPS* preoperative modified Glasgow Prognostic score; *POD* postoperative day.

The relationship between each anaesthetic group and POD 2 CRP > 150 mg/L of patients undergoing elective open surgery for colon cancer is shown in Table 4. There was a significant association between anaesthetic technique in particular, general + epidural (p = 0.02) and general + spinal (p = 0.01) with POD 2 CRP > 150 mg/L.

**Table 4:** The relationship between each anaesthetic technique and POD 2 CRP of patients undergoing elective open surgery for colon cancer.

Group of anaesthesia	Number of patients	Adjusted Z Score	P-value
General alone	15	1	0.15
General + Epidural	89	2.2	0.02
General + TAP-block	10	-0.5	0.58
General + Local infiltration	3	-1.4	0.15
General + Spinal	16	**-**2.5	0.01

## 5. Discussion

In this retrospective observational study, there was a significant association between type of anaesthesia, and the magnitude of the postoperative day 2 CRP in patients who underwent open but not laparoscopic surgical resection for colorectal cancer. There was a reduction in patients with POD 2 CRP >150 mg/L in the GA+TAP, GA+LA and GA+Sp groups. Patients receiving GA + epidural seemed more likely to have a POD 2 CRP >150 mg/L. Although a number of confounding factors were examined, this may reflect a higher risk patient cohort and confounding by indication. The exact nature of the relationship between type of anaesthesia and post-operative SIR remains unclear and requires further investigation. However, it is clear that the type of anaesthesia is secondary to the effect of surgical approach, in particular laparoscopic surgery on the postoperative SIR.

In the present study, the association between anaesthesia type and post-operative SIR in open colorectal surgery provides new information in an area of clinical uncertainty. To our knowledge, few studies have examined the effect of specific anaesthetic techniques on the post-operative SIR. In particular, the effect of anaesthesia on the post-operative CRP concentration is not clear. Chen and co-workers (2015), in 53 patients undergoing resection of colon cancer, reported a significant reduction on day 2 post-operative CRP with general plus epidural anaesthesia compared with general anaesthesia. However, it was not clear whether an open or laparoscopic surgical approach was used [13]. Papadima and co-workers, in 40 patients receiving open surgery for colon cancer, reported a decrease of post-operative day 2 CRP in patients receiving general anaesthesia compared with epidural analgesia [14]. In contrast, Gasiunaite and co-workers, in 53 patients receiving laparoscopic colorectal resection, reported no significant difference in the post-operative CRP concentration on day 2 and 3 in patients receiving general anaesthesia versus general plus epidural anaesthesia [15]. Taken together with the present results in 409 patients and given that the magnitude of the postoperative SIR is greater in open surgery [3], it may be that regional anaesthetic techniques have a greater potential to modulate the magnitude of the postoperative SIR.

The anaesthetic technique varied with time with a notable increase in spinal opioid analgesia and general anaesthesia without regional analgesia. This is consistent with the evolution of anaesthesia according to ERAS principles. The benefits of epidural analgesia are less apparent in the ERAS setting and may even be disadvantageous in its association with hypotension, urinary retention, failure rates and rare but serious complications such as epidural haematoma and abscess. However, epidural anaesthesia is still recommended in high risk patient groups, patients with chronic pain and those considered likely to convert to an open procedure [16, 17].

In addition to central neuraxial blockade, other regional anaesthetic techniques including TAP-block or local anaesthetic infiltration, can be used for postoperative pain management in abdominal surgery. A renewed interest in the use of abdominal wall blocks has resulted in a large number of studies examining pain scores and the consumption of opioids after surgery. TAP block remains the most studied of these techniques though evidence remains heterogeneous and questions remain as to the optimal technique, method of administration, dosage and efficacy in different types of surgery. TAP blocks are recommended by ERAS guidelines in the performance of minimally invasive colorectal surgery [10]. However, their effect on the post-operative SIR remains to be defined.

Preoperative adjuvants such as the intravenous administration of dexamethasone, are commonly used in the anaesthetic practice to reduce the postoperative nausea and vomiting. Following abdominal surgery, preoperative use of dexamethasone may significantly reduce the magnitude of the postoperative SIR and postoperative complications [18, 19] though its potential immunosuppressive effects are as yet to be not determined. To date, dexamethasone has been considered as a part of fast track or enhanced recovery after surgery (ERAS) [6, 20]. Therefore, the administration of dexamethasone represents a potential confounder to any effects of anaesthesia type on the postoperative SIR.

Therefore, anaesthetic practices may vary widely within and across surgical approaches and even within enhanced recovery protocols [21]. Against this background it is difficult to speculate what anaesthetic regimen has the most profound effect on the postoperative SIR and what mechanism of action may be most efficacious to target to reduce the magnitude of the postoperative SIR. Therefore, it will require prospective examination of anaesthetic practice across multiple institutions to tease out the effects of anaesthesia on the postoperative SIR. Such work will provide the foundations of an evidenced based approach to developing an anaesthetic protocol to be used alongside existing enhanced recovery protocols.

Several limitations to this study need to be acknowledged. Firstly, this study includes patients from a single centre and is subject to the well-described limitations of retrospective analysis. For example, due to the detail of the data collected retrospectively, it was not possible to account for all the agents that may have been used in the provision of general anaesthesia and that may have influenced the postoperative SIR. Also, it was not possible to correct for all potentially confounding factors in the analysis. Therefore, further prospective work is required to examine the relationship between anaesthetic technique and the magnitude of the postoperative SIR in more detail.

## 6. Conclusion

In summary, in the largest study to date and in patients undergoing elective surgery for colon cancer, the anaesthetic approach may affect the magnitude of the postoperative SIR, as evidenced by post-operative CRP concentrations. Further prospective studies are required to confirm these findings.
